# Unexpected role of the L-domain of calpastatin during the autoproteolytic activation of human erythrocyte calpain

**DOI:** 10.1042/BSR20180147

**Published:** 2018-04-20

**Authors:** Roberta De Tullio, Alice Franchi, Antonino Martines, Monica Averna, Marco Pedrazzi, Edon Melloni, Bianca Sparatore

**Affiliations:** 1 Department of Experimental Medicine (DIMES), Biochemistry Section, University of Genova, Viale Benedetto XV-1, 16132 Genova, Italy; 2 Centre of Excellence for Biomedical Research (CEBR), University of Genova, Viale Benedetto XV-7, 16132 Genova, Italy

**Keywords:** Calpastatin L-domain, Calpain-1 autoproteolysis, calpain/calpastatin system

## Abstract

Autoproteolysis of human erythrocyte calpain-1 proceeds *in vitro* at high [Ca^2+^], through the conversion of the 80-kDa catalytic subunit into a 75-kDa activated enzyme that requires lower [Ca^2+^] for catalysis. Importantly, here we detect a similar 75 kDa calpain-1 form also *in vivo*, in human meningiomas. Although calpastatin is so far considered the specific inhibitor of calpains, we have previously identified in rat brain a calpastatin transcript truncated at the end of the L-domain (cast110, L-DOM), coding for a protein lacking the inhibitory units. Aim of the present study was to characterize the possible biochemical role of the L-DOM during calpain-1 autoproteolysis *in vitro*, at high (100 µM) and low (5 µM) [Ca^2+^]. Here we demonstrate that the L-DOM binds the 80 kDa proenzyme in the absence of Ca^2+^. Consequently, we have explored the ability of the 75 kDa activated protease to catalyze at 5 µM Ca^2+^ the intermolecular activation of native calpain-1 associated with the L-DOM. Notably, this [Ca^2+^] is too low to promote the autoproteolytic activation of calpain-1 but enough to support the catalysis of the 75 kDa calpain. We show for the first time that the L-DOM preserves native calpain-1 from the degradation mediated by the 75 kDa form. Taken together, our data suggest that the free L-domain of calpastatin is a novel member of the calpain/calpastatin system endowed with a function alternative to calpain inhibition. For this reason, it will be crucial to define the intracellular relevance of the L-domain in controlling calpain activation/activity in physiopathological conditions having altered Ca^2+^ homeostasis.

## Introduction

The Ca^2+^-dependent proteolytic system is composed by a family of proteases, named calpains, and by their specific inhibitors calpastatins. The best characterized proteases are calpain-1 and -2 (also named as µ- and m-calpains) that require μM and mM Ca^2+^ concentration for their catalytic activity *in vitro*, respectively [1,2]. In normal cell conditions, following an increase in [Ca^2+^]_i_, calpain-1 catalyzes the limited digestion, rather than the degradation, of specific proteins leading to changes of their physiological functions [2]. Since calpain-1-mediated proteolysis influences several cell activities, such as migration, proliferation, and apoptosis [3,4], the activation/activity of the protease requires a fine modulation to prevent abnormal degradation of its targets.

In addition to [Ca^2+^], calpain is regulated by calpastatin that, in its full length version, includes an N-terminal region without inhibitory activity, followed by four repetitive inhibitory domains that can be liberated by calpain-dependent limited digestion [5]. This conservative processing of calpastatin induces an increase in the intracellular amount of forms endowed with calpain inhibitory activity and allows an efficient control of activated calpain [5,6]. However, it has been demonstrated that in conditions of persistently altered Ca^2+^ homeostasis, calpain-1 can exert a pathological function through an extensive intracellular degradation of specific proteins [7]. In diseases characterized by Ca^2+^ dysregulation, overexpression of calpastatin inhibitory units successfully prevents this excessive digestion of calpain-1 targets [8,9].

Extensive information, also collected from transgenic animal models, is available on the structural and functional properties of the calpastatin inhibitory units, whereas the role of the upstream N-terminal region remains poorly explored. Importantly, while the four inhibitory units show high homology amongst each other, the N-terminal domain undergoes alternative splicing. In fact, four types of calpastatin have been identified depending on the first exon translated [10]. Calpastatin type I contains the XL-L regions in the N-terminal domain and starts with exon 1xa, type II differs from type I for the first exon translated (1xb), whereas type III lacks the XL-region as it starts in exon 1u which is upstream of exon 2, the first exon of the L-domain. Finally, type IV calpastatin contains only the four inhibitory domains and is tissue specific [11]. A similar calpastatin splice variant has been also found in rat brain (castATG873) [12]. Upstream of exons 1xa, 1xb, 1u, the calpastatin gene contains three promoters sensitive to a variety of inducers that can modulate the expression of calpastatin forms with a peculiar XL domain [13,14]. Moreover, alternative splicing inside the L-domain promotes the generation of other forms of calpastatin not yet functionally characterized [12,15]. Specifically, human calpastatin can undergo splicing at exons 3 and/or 5 [10,16,17]. Splicing at exon 3 was also observed in porcine calpastatin exclusively in skeletal muscle, while heart contains transcripts that include or lack exon 3 [18]. Moreover, we have shown previously that Ca^2+^- or cAMP-dependent phosphorylation of calpastatin in exon 6 of the L-domain can regulate the intracellular localization of the inhibitor [19]. These data demonstrated that the L-domain contributes to the modulation of calpain inhibition by allowing or avoiding the enzyme–inhibitor interaction.

Amongst the various splice forms of calpastatin at the L-domain, we have previously identified in rat brain a transcript (RNCAST110, GenBank Y13590) that consists of exons 2, 4–6, and the beginning of exon 7 [20]. This calpastatin species (calpastatin free L-domain, corresponding to cast110 (GenBank Y13590) (L-DOM)), that does not contain any inhibitory domain, cannot be distinguished in tissues from fragments containing the N-terminal region, and derived from calpain-mediated proteolysis of full-length calpastatins [12]. Hence, we have previously produced the L-DOM as a recombinant protein and we have demonstrated that the presence of L- or XL-L-domains in calpastatin molecules is responsible for the loss of inhibitory efficiency observed at concentrations of calpastatin that would be expected to give complete calpain inhibition [21]. It is important to consider that the L-domain of calpastatin can interact with calpain also in the absence of Ca^2+^ when the protease is in its inactive conformation [22]. Conversely, the interaction of the inhibitory unit only occurs in the presence of Ca^2+^ when the protease is activated by realignment of the catalytic triad [23].

Here we have explored the role of the L-domain in the process of calpain-1 activation *in vitro*.

## Materials and methods

ECL® Select Detection System was purchased from GE Healthcare (Milan, Italy). Anti-mouse IgG HRP-linked (1:5000) was from Cell Signaling (Danvers, MA). mAb to the catalytic subunit of calpain-1 was from Sigma–Aldrich (St. Louis, MO, U.S.A.).

The meningothelial meningioma samples were obtained from the Neurosurgery Department of the IRCCS-AOU San Martino IST, (Genova, Italy) after patients’ informed consent and Institutional Ethical Committee approval. The patients underwent surgery for the first time and never received chemo- or radiotherapy. Pathological analysis classified meningothelial meningiomas as grade I according to World Health Organization criteria [24]. The tissue specimens collected in the operation room were quickly frozen in liquid nitrogen, and stored at −80°C until processing.

### Purification of calpain-1 from human erythrocytes and assay of proteolytic activity

Human erythrocyte calpain-1 was purified to homogeneity as described in [25]. The specific activity of the purified enzyme was 650 units/mg. One unit of calpain activity is defined as the amount of enzyme required to release 1 µmol/h of free α-amino groups following 10-min incubation at 25°C, in the presence of 50 mM sodium borate buffer pH 7.5, 1 mM CaCl_2_, and 2 mg/ml acid-denatured human globin [26] as substrate. The amount of amino groups liberated as acid soluble peptides was measured with fluorescamine [27]. The purified protease was stored at 4°C in 50 mM sodium borate buffer pH 7.5 containing 0.1 mM EGTA and 0.5 mM 2-mercaptoethanol (Buffer A).

### Preparation of crude protein extracts from human meningothelial meningiomas and Western blotting

Tissue samples (25 mg) were defrosted, washed twice in chilled PBS (137 mM NaCl, 2.7 mM KCl, 10 mM Na_2_HPO_4_, NaH_2_PO_4_ pH 7.4), roughly minced and suspended in 500 μl of ice-cold 50 mM sodium borate buffer, pH 7.5, containing 1mM EGTA and Pierce™ Protease Inhibitor Tablets, EDTA-free (lysis buffer). Samples were homogenized with a Waring Blender homogenizer at half speed (four cycles of 10 s), and lysed by sonication (three bursts of 10 s) in an ice bath. The homogenates were centrifuged at 400×***g*** for 5 min at 4°C, the particulate material was discarded and the protein content was determined on the supernatant (crude lysate) according to the Lowry method. Crude lysates (10 μg/lane) were submitted to 10% SDS/PAGE and proteins were then transferred to nitrocellulose membranes (Bio-Rad, Hercules, CA, U.S.A.) by electroblotting. The membranes were probed with calpain-1 antibody, the immunoreactive signals were then probed with a peroxidase-conjugated secondary antibody and detected with ECL® Select (GE Healthcare, Milan, Italy) by using a Bio-Rad ChemiDoc™ XRS apparatus.

### Preparation of recombinant calpastatins

**Scheme 1:**
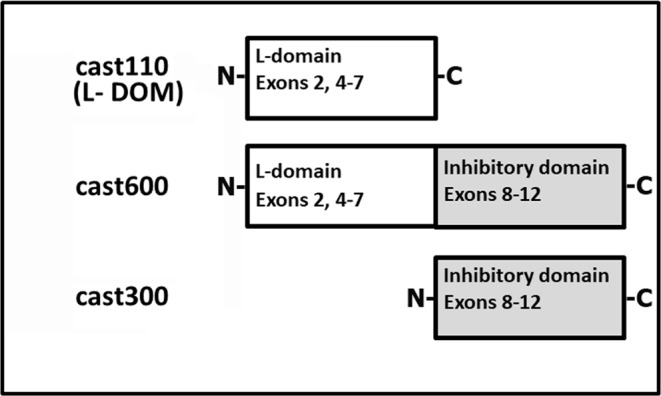
Simplified representation of the three recombinant calpastatin species used in the present paper These calpastatin species are spliced forms lacking exon 3 [41]. Cast110 recombinant protein corresponds to the calpastatin transcript detected in rat brain and having the GenBank accession number: Y13590, RNCAST110.

RNCAST300 (cast300), RNCAST600 (cast600), and RNCAST110 (cast110, calpastatin L-DOM, GenBank Y1359) proteins (see also Scheme 1) were prepared as described in [28]. Purified recombinant calpastatins were heated for 5 min at 95°C and stored at 4°C in Buffer A.

### Immunoprecipitation of calpastatin

Recombinant calpastatins (220 pmol) were separately mixed with purified human erythrocyte calpain-1 (70 pmol) in a final volume of 100 µl of Buffer A. Samples were placed in an end-over-end rotator for 1 h at 25°C before adding 2 µg of calpain-1 antibody. The mixtures were incubated overnight at 4°C with gentle rotation, and the day after Protein G-Sepharose (40 µl, equilibrated in Buffer A) was added to each sample. These mixtures were rotated end-over-end for 2 h at 4°C and then centrifuged for 5 min at 400×***g***. The supernatant (output) was lyophilized, dissolved in 30 µl SDS/PAGE loading solution (60 mM Tris/HCl pH 6.8 containing 2% SDS, 2% 2-mercaptoethanol, and 10% glycerol), and heated for 5 min at 95°C. The Sepharose beads were washed three times with 500 µl Buffer A, and suspended in 30 µl SDS/PAGE loading solution. As a control of the electrophoretic mobility of the recombinant calpastatins in SDS/PAGE, 220 pmol of each calpastatin were diluted in 140 µl Buffer A, and treated as the output samples. The samples were run on 12% SDS/PAGE followed by gel staining with Coomassie Brilliant Blue.

### Overlay assay

Increasing amounts of cast300, cast600, and cast110 (0.5, 1, 2 pmol) were spotted on to a nitrocellulose membrane. The membrane was incubated in blocking buffer (5% skim milk, dissolved in PBS containing 0.1% Tween 20 (T-PBS)) for 1 h at 25°C, washed twice in T-PBS and twice in PBS. The membrane was equilibrated by three washes, 10 min each, in buffer A and then incubated with purified calpain-1 (390 pmol in 5 ml Buffer A), with gentle agitation for 2 h at 25°C. The membrane was then washed twice in Buffer A without 2-mercaptoethanol, and probed (overnight 4°C), with calpain-1 antibody diluted in T-PBS. The presence of the calpain-1/calpastatin complex was detected using a peroxidase-conjugated secondary antibody (2 h incubation, with gentle agitation, 25°C). The immunoreactive signals were developed with ECL® Select and detected with a Bio-Rad ChemiDoc™ XRS apparatus.

### Immunoblot analysis on purified proteins

Samples of each incubation mixture were submitted to SDS/PAGE (10%), and proteins were then transferred to nitrocellulose membranes (Bio-Rad, Hercules, CA, U.S.A.) by electroblotting. To detect erythrocyte calpain-1, nitrocellulose membranes were probed with a calpain-1 antibody, followed by a peroxidase-conjugated secondary antibody. The immunoreactive signals were developed with ECL® Select, detected with a Bio-Rad ChemiDoc™ XRS apparatus, and quantitated using the Quantity One 4.6.1 software (Bio-Rad).

### Autoproteolysis of human erythrocyte calpain-1

Purified human erythrocyte calpain-1 (5 pmol, in 80 µl) was incubated in 50 mM sodium borate buffer pH 7.5, containing 100 μM Ca^2+^ at 30°C. At 0, 5, 10, 20 min, aliquots (25 µl) were collected, mixed with 5 µl SDS/PAGE loading solution 6×, heated for 5 min at 95°C, and 20 µl were submitted to SDS/PAGE (10%). Calpain-1 was detected by immunoblotting as described above.

### Intermolecular activation of calpain

To obtain the 75 kDa activated calpain-1, purified human erythrocyte calpain (50 pmol, in 800 µl) was incubated in 50 mM sodium borate buffer pH 7.5, containing 100 μM Ca^2+^ for 3 min at 30°C. Aliquots of 220 µl (containing 14 pmol of activated calpain-1) were collected, the [Ca^2+^] was rapidly lowered to 5 µM (final concentration) by adding a mixture containing inactive calpain (50 pmol, in 800 µl) in the absence or presence of the L-DOM (10 pmol). Since both calpain and calpastatin are stored in Buffer A that contains 0.1 mM EGTA to avoid calpain activation, the association of the L-DOM to native calpain occurs in the absence of Ca^2+^ [22]. At the times indicated, aliquots of 80 µl were used to measure the activity of calpain both in 5 µM Ca^2+^ and in 1 mM Ca^2+^ (final concentration) in the presence of globin as a substrate, while aliquots of 25 µl were submitted to SDS/PAGE (10%). Calpain-1 was detected by immunoblotting as described above.

## Results

### Conversion 80 kDa→75 kDa calpain-1 *in vitro* and *in vivo*

**Figure 1:**
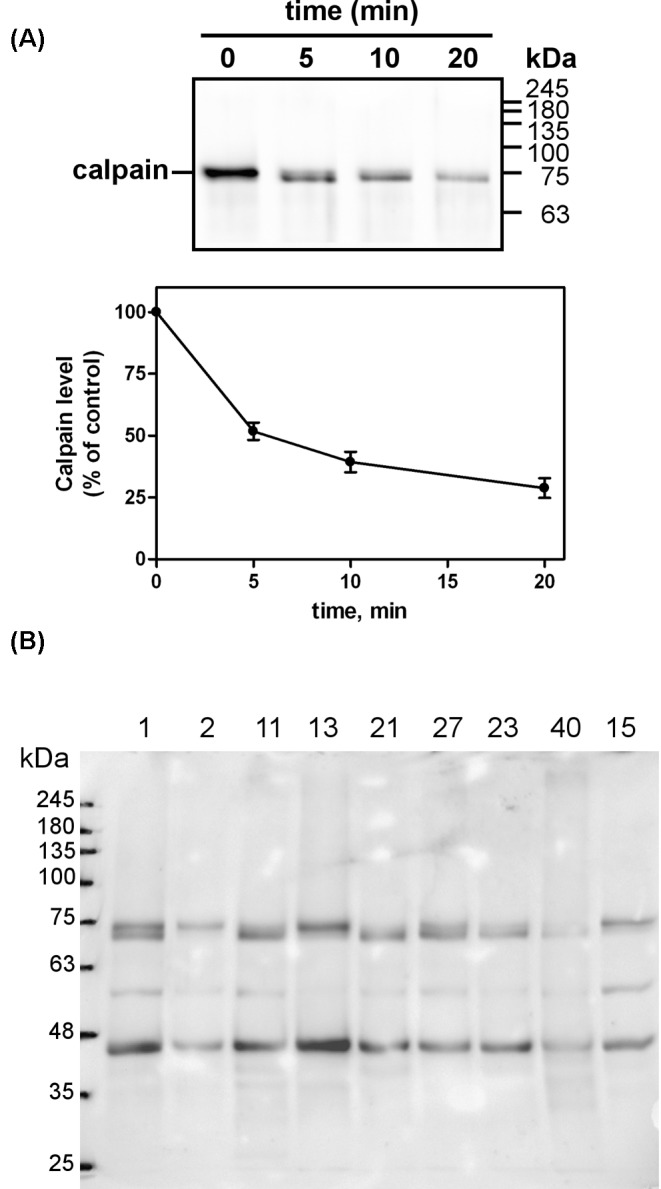
Autoproteolysis of human erythrocyte calpain-1 (**A**) Purified calpain-1 (5 pmol, 80 µl) was incubated in 50 mM sodium borate buffer pH 7.5, containing 100 μM Ca^2+^ at 30°C. At the times indicated, aliquots (25 µl) were collected, and 5 µl of Laemmli buffer 6× were added. The samples were heated for 3 min at 95°C and submitted to SDS/PAGE (10%). Calpain-1 protein was detected by immunoblotting and the relevant band was quantitated as described in ‘Materials and methods’ section. Data presented are mean ± S.E.M. of three independent experiments. (**B**) Crude lysates (10 µg) prepared from human meningothelial meningiomas samples (see ‘Materials and methods’ section) were submitted to SDS/PAGE (10%) and calpain-1 was detected by immunoblotting.

We have used in all experiments purified human erythrocyte calpain which belongs to the calpain-1 family and requires for its activation *in vitro* aproximately 15–25 µM Ca^2+^ [29]. Exposure to Ca^2+^ ions induces a conformational change in the protease finally leading to the removal of a 5-kDa fragment from the N-terminal region of the catalytic subunit, and the conversion from an 80 kDa proenzyme into a 75 kDa activated enzyme. The activated protease requires *in vitro* 5–6 µM Ca^2+^ to display proteolytic activity in the presence of substrate [29–32]. Indeed, as shown in Figure 1A, when human erythrocyte calpain-1 was exposed to 100 µM Ca^2+^ without any exogenous substrate, the native 80 kDa catalytic subunit was converted into the 75 kDa form within 5-min incubation. Then this form slowly disappeared, and after 20 min, only a faint calpain immunoreactive band was still detectable.

A similar conversion occurs not only *in vitro*, at high [Ca^2+^] and in the absence of substrate, but also *in vivo*. As shown in Figure 1B, Western blotting of crude extracts from human meningothelial meningiomas probed with the same calpain-1 antibody used in Figure 1A, revealed that six samples out of nine contained both the 80 and the 75 kDa forms of calpain-1. Specifically, the samples from patients 2, 13, and 15 contained mostly native 80 kDa calpain-1, while the other samples showed different amounts and different ratios of the 80/75 kDa forms. These results indicate that this processing of calpain-1 is also detectable inside the cell where calpain-1 is at physiological Ca^2+^ and surrounded by many possible protein targets. Moreover, the presence of discrete immunoreactive fragments derived from the further degradation of the catalytic subunit, confirms that calpain-1 undergoes normally a proteolytic turnover with the production of active and inactive fragments. Importantly, to block any cell protease activity, we carried out the preparation of the crude extracts in the presence of a cocktail of protease inhibitors (see ‘Materials and methods’ section).

### Conversion 80 kDa→75 kDa calpain-1 in the presence of cast600, cast300, and cast110

**Figure 2:**
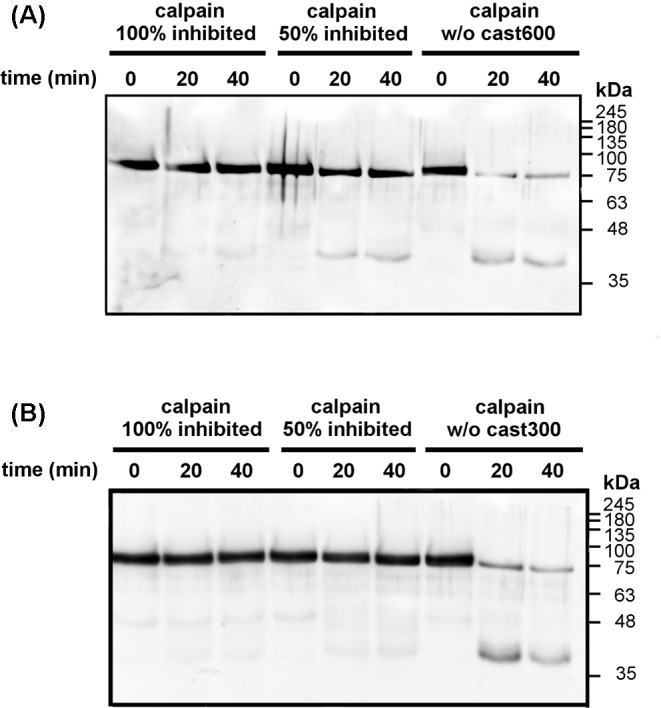
Autoproteolysis of calpain-1 in the presence of recombinant cast 600 and cast 300 Purified calpain-1 (5 pmol, 80µl) was incubated in 50 mM sodium borate buffer pH 7.5, containing 100 μM Ca^2+^ at 30°C in the absence or in the presence of cast600 (**A**) or cast300 (**B**). At the times indicated, aliquots (25 µl) were collected, and submitted to SDS/PAGE (10%) followed by immunoblotting to detect calpain-1 as specified in the legend to Figure 1. The two recombinant calpastatins were in amounts (3 and 6 pmol, respectively) causing either 50% inhibition of calpain activity or 100% theoretic inhibition [21]. Inhibition of calpain was calculated by measuring residual calpain activity in 1 mM Ca^2+^, in the presence of increasing amounts of the recombinant calpastatin, and globin as substrate (see also ‘Materials and methods’ section). Similar results have been obtained in two additional experiments done by using a different preparation of recombinant calpastatins.

To establish the possible role of the inhibitory and L-domain of calpastatin during the production of the 75 kDa calpain form *in vitro*, we carried out autoproteolysis of calpain-1 in the presence of cast110, cast600, and cast300 recombinant calpastatins (see Scheme 1). Except for the single inhibitory unit (cast300), that we produced deliberately to dissect the functional characteristics of different calpastatin domains, the transcripts for both RNCAST110 (cast110) consisting only of the L-domain (L-DOM) and RNCAST23 (cast600) also including the first inhibitory unit, have been identified in rat brain [20]. As shown in Figure 2, saturating amounts of both cast600 (Figure 2A) and cast300 (Figure 2B) prevented the conversion of calpain-1 into the 75 kDa form. Indeed, even after 40 min exposure to 100 µM Ca^2+^ the intensity of the 80 kDa band of calpain-1 remained nearly the same.

When the amounts of the two calpastatins were halved, the 75 kDa calpain became detectable. However, while cast300 was still effective in inhibiting the autoproteolysis of calpain-1, cast600 only partially protected the enzyme from digestion and the decrease in the 75 kDa form was less pronounced than that observed at the same times of incubation without inhibitor. Consequently, calpain-1 discrete fragments accumulated when cast600, but not cast300, was in amounts able to inhibit 50% of calpain-1 activity (Figure 2B). As reported previously, the 75 kDa form undergoes further degradation to discrete fragments of approximately 46 and 34 kDa, respectively [29,33]. While we detected the 46 kDa fragment of calpain-1 in the crude lysates from meningiomas (see Figure 1B), the lower Mr species was produced only *in vitro*, where activation of calpain-1 proceeds in the absence of any other substrate, except for calpain-1 itself, and at a non-physiological [Ca^2+^]. Since cast600, differently from cast300, can bind calpain-1 both through the L-domain and the inhibitory unit [21], it is conceivable to hypothesize that the L-domain can interfere with the efficient insertion of the inhibitory domain in the catalytic cleft [21]. In this condition, the catalytic cleft is still available for autolysis and consequently for activation of the protease [30].

**Figure 3:**
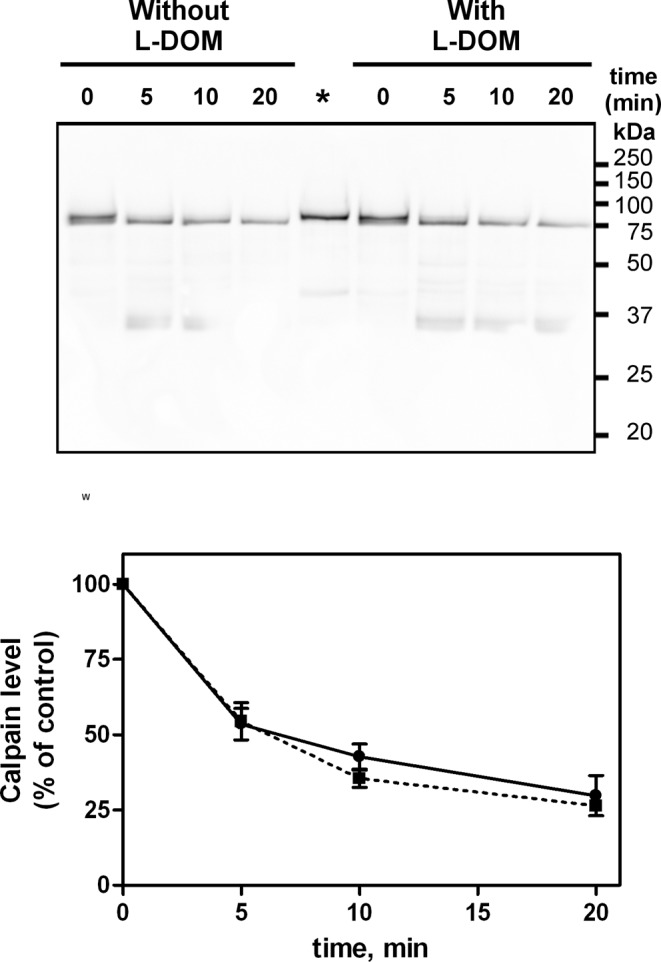
Autoproteolysis of calpain-1 in the presence of the L-DOM Purified calpain-1 (10 pmol, 160 µl) was incubated in 50 mM sodium borate buffer pH 7.5, containing 100 μM Ca^2+^ at 30°C in the absence or presence of the L-DOM (3.5 pmol). At the times indicated, aliquots (25 µl) were collected and submitted to SDS/PAGE (10%) followed by immunoblotting to detect calpain-1 (upper panel). The relevant band was quantitated as described in ‘Materials and methods’ section (lower panel): (•) continuous line, without L-DOM and (▪) dotted line, with L-DOM. Data presented are mean ± S.E.M. of four independent experiments. * Native calpain-1 (1 pmol) was loaded as a control.

Next, we analyzed calpain-1 activation in the presence of the free L-DOM (cast110), a condition in which the catalytic cleft is not engaged by the inhibitory unit. As shown in Figure 3, calpain underwent conversion 80 kDa→75 kDa within 5-min exposure to 100 µM Ca^2+^ and discrete fragments of calpain were produced independently of addition of the L-DOM. The quantification of the 80/75 kDa level during the time course (Figure 3, lower panel) demonstrates that, in these conditions, the activation of calpain is not influenced by the presence of exogenous L-DOM.

### Ca^2+^-independent interaction of the L-DOM with native calpain-1

**Figure 4:**
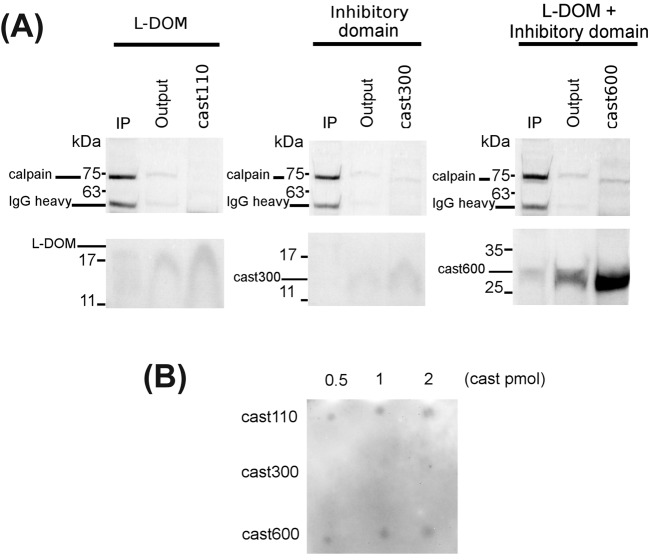
Association of cast110 (L-DOM) to calpain-1 in the absence of Ca^2+^ (**A**) IP of calpastatin: purified recombinant cast110 (L-DOM), cast300, and cast600 (220 pmol each) were incubated with human erythrocyte calpain-1 (70 pmol) in Buffer A as described in ‘Materials and methods’ section. The calpain/calpastatin complex was immunoprecipitated using calpain-1 antibody. Calpastatins were detected following SDS/PAGE (12%) and protein staining with Coomassie Brilliant Blue. (**B**) Overlay assay of calpastatins: increasing amounts of the three recombinant calpastatins cast110, cast300, and cast600 (0.5, 1.0, 2.0 pmol) were spotted on to a nitrocellulose sheet and purified human erythrocyte calpain-1 diluted in Buffer A was added (see ‘Materials and methods’ section). To target the calpain-1 associated with calpastatin, the same calpain-1 antibody as in (**A**) was used. One representative experiment of three (A) and four (B) is shown.

Our previous data demonstrated that only the calpastatin form containing the L-domain (cast600) and not cast300 can associate with calpain-1 in the absence of Ca^2+^, at a site proximal to the catalytic cleft [21,22]. To exclude the possibility that such Ca^2+^-independent binding, even if mediated by the L-DOM, needs the presence of the inhibitory unit to take place, we investigated whether cast110 *alone* could interact with calpain-1 in the absence of Ca^2+^. Since at present no antibodies are available to detect the L-DOM, we used two different approaches exploiting the specificity of the calpain-1 antibody as a marker of the binding. At first, we carried out immunoprecipitation (IP) of the three calpastatins species using the calpain-1 antibody and we detected the occurrence of the possible calpain/calpastatin complex following SDS/PAGE and Blue Coomassie staining. As shown in the lower panels of Figure 4A, while cast300 was only recovered in the output, the L-DOM was detected in the IP. Cast600, containing both the L-domain and the inhibitory domain, behaved as the L-DOM. We obtained similar results following an overlay assay where increasing amounts of the three calpastatin species were spotted on to nitrocellulose and then exposed to calpain-1 in the absence of Ca^2+^. The formation of the calpain-1/calpastatin complex was detected by using the calpain-1 antibody (Figure 4B). We observed that the Ca^2+^-independent interaction between calpain and calpastatin took place when the L-domain was both free (L-DOM, cast 110) or followed by the inhibitory domain (cast600). Since the absence of Ca^2+^ maintains calpain-1 inactive and its catalytic subunit in the native 80 kDa conformation, we next investigated whether the association of the L-DOM with the 80 kDa subunit could play a role in the intermolecular conversion 80 kDa→75 kDa mediated by activated 75 kDa calpain-1.

### Role of the L-DOM in the intermolecular 80 kDa→75 kDa conversion of calpain-1 mediated by activated calpain-1 at low Ca^2+^

**Figure 5:**
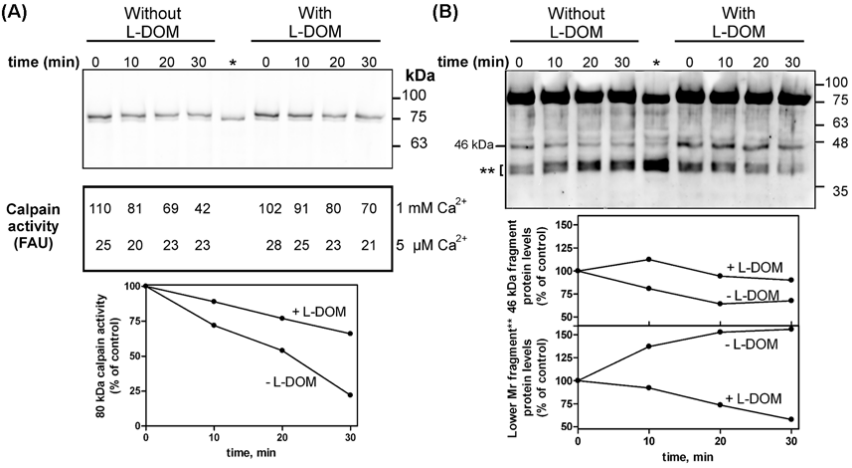
Intermolecular conversion of native 80 kDa into 75 kDa calpain-1 mediated by activated calpain-1 in the presence of L-DOM Purified human erythrocyte calpain-1 (50 pmol, 800µl) was first incubated in 50 mM sodium borate buffer pH 7.5, containing 100 μM Ca^2+^ for 3 min at 30°C to obtain the 75 kDa activated calpain-1. Then two aliquots (220 µl each) were collected, the [Ca^2+^] rapidly lowered to 5 µM by adding a mixture containing inactive calpain-1 (50 pmol, 800 µl) in the absence or presence of the L-DOM (10 pmol, molar ratio 80 kDa calpain-1/L-DOM, 5/1). (**A**) At the times indicated aliquots of 25 µl were submitted to SDS/PAGE (10%) followed by immunoblotting to detect calpain-1 (upper panel), while aliquots of 80 µl were used to measure the activity of calpain in 1 mM and 5 µM [Ca^2+^] in the presence of substrate (middle panel) (FAU, fluorescence arbitrary units). (Graph): the activity of the 80 kDa form was obtained by subtracting the activity of the 75 kDa form (fluorescence at 5 µM Ca^2+^) from the total calpain activity measured at 1 mM Ca^2+^ and is expressed as % of control (zero time). (**B**) Longer exposure of the same blot shown in (A). Quantitation of the immunoreactive signals corresponding to the 46 kDa fragment and to the lower Mr digestion products (**). Data are representative of four independent experiments showing similar results.

We prepared the 75 kDa calpain-1 by incubating the native enzyme in 100 µM Ca^2+^ for 3 min (the 80 kDa→75 kDa conversion reaches completion within 5 min, see Figure 3). Then the [Ca^2+^] was lowered to 5 µM and a sample of the 75 kDa calpain-1 was incubated with inactive 80 kDa calpain-1 which at 5 µM Ca^2+^cannot undergo activation (molar ratio 75 kDa/80 kDa calpain-1, 1/4) [1]. In this way, it is possible to establish if the active 75 kDa calpain can convert the inactive 80 kDa enzyme into the 75 kDa form at a Ca^2+^ concentration insufficient to promote the activation of native calpain-1. At a first sight (upper panel, left side of Figure 5A), it can be observed that while the intensity of the lower 75 kDa faint band remained nearly the same, the intensity of the upper 80 kDa band gradually decreased. This observation was supported quantitatively (see Figure 5A lower panel, left side and relevant graph, in Figure 5B) by the concomitant progressive decrease in the amount of the 46 kDa calpain-1 fragment (continuous line) and by the accumulation of lower Mr fragments. Aliquots of each incubation were also used to assay residual calpain-1 activity in the presence of substrate (Figure 5A). The decrease in the activity of calpain measured in 1 mM Ca^2+^ indicates that the 80 kDa calpain is directly degraded to inactive fragments [29,33]. The fact that the activity recovered at 5 µM Ca^2+^ never increased (the little activity detected deals with the 75 kDa calpain used in the incubation), confirms that in this condition no conversion 80 kDa→75 kDa occurred. Hence, at low Ca^2+^ concentrations the activated 75 kDa calpain-1 does not catalyze the calpain-1 conversion 80 kDa→75 kDa, but directly degrades the native enzyme to inactive fragments.

The same experiment was carried out in the presence of the L-DOM (molar ratio 80kDa calpain-1/L-DOM, 5/1). As shown in Figure 5A right side, the L-DOM partially protected native calpain-1 from the degradation mediated by the 75kDa form. Indeed, after 30 min incubation native calpain-1 retained 66% of its catalytic activity assayed in 1 mM Ca^2+^. At the same incubation time, in the absence of L-DOM, the 80 kDa residual activity was only 22% of the control (Figure 5A, bottom). In these conditions, the degradation of the 46kDa fragment was delayed and less pronounced than in the absence of L-DOM. Consequently, the lower Mr fragments did not accumulate (Figure 5B). Thus, at 5 µM Ca^2+^ the L-DOM exerts a protective action on native calpain-1 against the degradation promoted by activated calpain-1 molecules.

**Figure 6:**
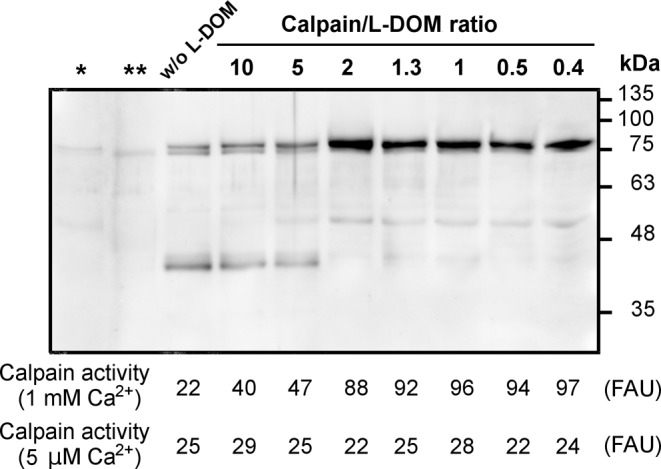
Intermolecular conversion of native 80 kDa into 75 kDa calpain-1 mediated by activated calpain-1 in the presence of increasing amounts of L-DOM Purified calpain-1 (15 pmol, 240 µl) was first incubated as in the legend to Figure 5 to obtain the 75 kDa activated calpain-1. Then aliquots (50 µl) were collected, the [Ca^2+^] rapidly lowered to 5 µM by adding a mixture containing inactive calpain (13 pmol, 210 µl) in the absence or presence of increasing amounts of the L-DOM. (Lower part of the figure): after 30-min incubation, 75 µl were used to measure the activity of calpain-1 in 1 mM and 5 µM [Ca^2+^] in the presence of substrate (see ‘Materials and methods’ section), and 25 µl were submitted to SDS/PAGE (10%). Calpain-1 was detected by immunoblotting as described in ‘Materials and methods’ section. As a control, an aliquot (25 µl) of calpain-1 before (*) and after 3 min activation in 100 µM Ca^2+^ (**) was submitted to SDS/PAGE (10%) followed by immunoblotting. The results shown in the figure are representative of three independent experiments. Abbreviation: FAU, fluorescence arbitrary units.

We finally explored whether the L-DOM could completely preserve the 80 kDa calpain-1 from the 75 kDa calpain-1-mediated degradation. To obtain this information, activated 75 kDa calpain-1 was incubated with native calpain-1 for 30 min at 5 µM Ca^2+^ in the presence of increasing amounts of L-DOM (molar ratio 75 kDa/80 kDa calpain, 1/4). As shown in Figure 6, at a 2/1 molar ratio 80 kDa calpain-1/L-DOM, the L-DOM was very efficient in preventing the degradation of native calpain-1. In any case, even at low concentrations of L-DOM the conversion 80→75 kDa did not occur, as at 5 µM Ca^2+^ we detected only the activity of the 75 kDa activated calpain-1 added to the incubation mixture. Moreover, the activity of the native calpain-1 assayed in 1 mM Ca^2+^ was completely preserved at a 1/1 molar ratio 80 kDa calpain-1/L-DOM.

## Discussion

We here report for the first time that the free L-domain of calpastatin (L-DOM), separated from the inhibitory units, protects at 5 µM Ca^2+^, native calpain-1 from the degradation catalyzed by activated calpain-1, *in vitro* and in the absence of substrate. This effect depends of the Ca^2+^-independent association between the L-DOM and the catalytic subunit of native 80 kDa human erythrocyte calpain-1. On the other hand, the L-DOM has no effect on the 80→75 kDa conversion of calpain-1 in 100 µM Ca^2+^. Thus the L-DOM differently affects the processes of activation and inactivation of calpain-1, depending on the amount of Ca^2+^ present. In the condition of ‘high’ Ca^2+^, the presence of the inhibitory unit in the catalytic cleft (experiments with cast300 and cast600) delays calpain-1 activation, but cast600, also containing the L-DOM, is less efficient than cast300. This result is in agreement with our previous data concerning the loss of inhibitory efficiency at high calpastatin concentrations, observed for the inhibitor species containing this N-terminal non-inhibitory region [21]. Since the L-domain of calpastatin binds calpain-1 at a site close to the catalytic cleft, and the inhibitory domain can bind both to the catalytic cleft and other sites in the EF-regions of calpain domains IV and VI [34–37], cast600, because of the L-domain, can hamper the entrance of the inhibitory unit in the catalytic cleft. As a result, the catalytic site can be occupied by the substrate, even in the presence of high amounts of calpastatin. Thus, the L-domain of calpastatin can regulate both the efficiency of the downstream inhibitory units and the activation process of calpain-1.

The autoproteolytic activation of calpain-1, leading to the production of a 75 kDa active form, is still under investigation. Indeed, structural studies indicate that neither autolysis nor subunit dissociation seem necessary to activate the protease [38,39]. In these studies, the authors use recombinant calpain constructs composed by hybrids of the m-calpain (calpain-2) catalytic subunit and a truncated form of the small subunit of calpain [38]. Interestingly, Tompa et al. [40] have carried out a similar experiment concerning the *intermolecular* activity of calpain-1. They demonstrate that following activation, human erythrocyte calpain-1 can activate m-calpain by fragmenting its small subunit. At present, we do not know the dynamics of the processing of the small subunit of erythrocyte calpain in our experimental conditions, because here we use an antibody directed toward the catalytic subunit of calpain-1. Our current and future investigations aim to explore the possible role of the small subunit during the intermolecular degradation of native calpain mediated by the low Ca^2+^ -requiring 75 kDa form. This aspect will be analyzed both in the presence and in the absence of the L-DOM.

Regarding the production of the 75 kDa form of calpain-1, we think that this is the result of an autoproteolytic event promoted by conformational changes induced by Ca^2+^. We exclude that this is an experimental artifact caused by the absence of exogenous substrates or by the high calpain-1 and Ca^2+^concentrations used *in vitro*. This conclusion is supported by the identification of the 80 and 75 kDa calpain-1, as well as of lower Mr calpain-1 species also *in vivo*.

This fact not only indicates that the 75 kDa calpain-1 is produced at intracellular Ca^2+^ concentrations, but also that the accumulation of discrete fragments of calpain-1 is part of a normal intracellular processing of this enzyme.

Alignment of the N-terminal region of human calpain-1 and calpain-2 sequences shows the occurrence of low homology. Specifically, calpain-1 is characterized by an N-terminal 10 amino acid residues extension. This region, possibly involved in the autolytic process, has not been included in the crystallographic studies concerning the µ-like calpain form [23]. Anyway, our data based on changes of molecular and catalytic properties of calpain-1, demonstrate that at low Ca^2+^, the 75 kDa form is able to recognize as a target and catalyze the degradation of the native 80 kDa calpain-1 molecules into inactive fragments.

Here, we have also demonstrated that not only cast600, endowed of both the L-domain and the inhibitory unit, but also the isolated L-domain bind to native erythrocyte calpain-1 in the absence of Ca^2+^, when the protease is in its inactive conformation. We have exploited this property of the L-DOM to investigate its possible role on the intermolecular processing of calpain-1 molecules. To this purpose, we have used 5 µM Ca^2+^, an amount of the metal ion too low for the activation of calpain-1, but sufficient for the catalytic activity of the 75 kDa form. In these conditions, the L-DOM protects the native 80 kDa calpain-1 from the proteolytic degradation mediated by the 75 kDa form. In fact, the activity of the 80 kDa calpain, assayed at 1 mM Ca^2+^ on an exogenous protein target, results completely preserved. Hence, the native calpain-1, instead of being directly degraded by the activated 75 kDa calpain, remains fully functional with the catalytic cleft available for substrate digestion.

It would be also interesting to explore the protective effect played by calpastatins containing both the L-domain and the inhibitory units, but we will address this aspect using a different strategy. Indeed, the inhibitory units of these calpastatins, by exploiting their L-domain, would engage the catalytic cleft of the native calpain molecules in the absence of Ca^2+^, prior addition of the 75 kDa activated calpain-1. Moreover, since it is well known that the inhibitory unit binds to calpain only in the presence of Ca^2+^ we cannot exclude that this calpastatin could inhibit the activated calpain-1 when it is added to the native enzyme together with Ca^2+^. As a result, in these conditions, it is not possible to distinguish between the residual activity of the native calpain-1 ascribed to the L-DOM, from that depending of the inhibitory units.

In conclusion, our new observations indicate that the splice variant of rat calpastatin that only contains the L-domain can play a modulatory role on the amount of calpain-1 molecules that could be either activated or directly degraded before activation occurs. Further investigations are in progress (i) to assess if a similar spliced transcript for calpastatin is present in humans; (ii) to establish the presence of the relevant protein translated from this transcript. This aspect is still difficult to address since calpastatin undergoes calpain-mediated proteolysis that produces different fragments, including the possible liberation of free L-domains; (iii) to obtain a specific antibody directed towards the L-DOM; (iv) to study the function of the L-DOM *in vivo* by transfection experiments on eukaryotic cells.

## Abbreviations

IP: immunoprecipitation
HRP: horse radish peroxidase
EF: EF-hand
L-DOM: calpastatin free L-domain, corresponding to cast110 (GenBank Y13590)
T-PBS: PBS containing 0.1% Tween 20
